# Helminths and Bacterial Microbiota: The Interactions of Two of Humans’ “Old Friends”

**DOI:** 10.3390/ijms232113358

**Published:** 2022-11-01

**Authors:** Kevin Llinás-Caballero, Luis Caraballo

**Affiliations:** Institute for Immunological Research, University of Cartagena, Cartagena 130014, Colombia

**Keywords:** helminths, microbiota, developing countries, diversity, composition

## Abstract

Humans have coexisted with helminths and bacteria for the entire existence of our species. Nowadays, helminth infections affect more than 1.9 billion people worldwide, especially in underdeveloped regions that lack optimal sanitary conditions. In addition, commensal microorganisms inhabit several compartments of humans, including the gastrointestinal tract, constituting what we know as the microbiota. Helminths and bacterial microbiota can interact in various ways. In this review, the interactions between helminths and commensal bacteria are analyzed in both animal models and humans. In developing countries, the gut microbiota exhibits high diversity, which could be linked to the high burden of helminthiasis in these areas. In fact, several studies show that helminth infections are associated with an increased gut microbiota diversity and changes in its composition. Interestingly, these changes can modify the risk for some diseases, such as asthma, colitis, viral infections, and metabolic conditions. Besides, the microbiota is necessary for the establishment of some helminth infections and can also influence the evolution of these diseases. Specific bacterial taxa can contribute to the resistance or susceptibility to certain helminths. The mechanisms underlying helminth–microbiota interactions are not completely understood. More research is necessary to address this and other unmet needs, especially considering that available studies are heterogeneous and sometimes yield conflicting results.

## 1. Introduction

Since birth, humans are colonized by a diversity of bacteria in several tissues such as the skin, the gastrointestinal tract, and the respiratory tract, forming a bacterial community that, together with other microorganisms such as fungi, protozoa, and viruses, make up the microbiota. This review will focus on the bacterial component of the microbiota and this term will be used hereon to refer to commensal bacterial communities. Host genetics influence the structure of the microbiota. Some genetic variants have been associated with the diversity and composition of microbiota in humans [1,2,3,4,5], and together with environmental factors such as delivery mode, breastfeeding, diet, and lifestyle, shape the human microbiota throughout life. In addition, viruses, fungi, and parasites (those “old friends” [6]) can also modify it. For instance, in the gut, bacteria can coexist with intestinal helminths (e.g., *Ascaris lumbricoides*, *Trichuris trichiura*, *Necator americanus*), establishing particular interactions that could be important in regions where helminth intestinal infections are endemic. In part because microbiota data from these regions are scarce and underrepresented in comparison with those from wealthy countries [7], the relationships between intestinal helminths and microbiota are poorly understood. However, considering the importance of both groups of organisms on several aspects of human physiology, including the immune responses, it is necessary to further explore their potential interactions.

The influence of microbiota on health and diseases, and particularly on the immune system, has been increasingly studied, revealing interesting microbiota-mediated axes [8]. Likewise, the influence of helminthiasis, such as ascariasis, on immune conditions (e.g., respiratory allergies) has been also described [9,10], and possible consequences of the interactions between microbiota/helminths on immune responses have been reported [11]. In this study, the scientific literature about the interactions between helminth infections/intestinal microbiota and their potential impact on health and disease was reviewed, including studies in humans and animal models. Even though there are interesting relationships between some components of the gut microbiota and intestinal parasites, this review focused on helminths and bacteria.

## 2. Characteristics of the Microbiota in Individuals from Low- and Middle-Income Countries

According to the latest World Bank classification of the world’s economies, countries with a gross national income of USD 13,205 or less are considered as low and middle-income countries (LMICs) [12]. In 2020 more than 6.5 billion people live in LMICs [13], the majority of the world’s population. These nations are usually affected by poor hygiene and sanitation conditions, undernutrition, lack of safe drinking water, high rates of infectious diseases (including neglected tropical diseases such as soil-transmitted helminthiasis), air and water pollution, and substandard access to healthcare, all of them with significant impacts on the physiology and health status of the population. Even within countries, some people are at a higher risk of dealing with these challenges throughout their life than others due to wealth inequality. Then, it is reasonable to think that the microbiota of those who live in LMICs or have lower socioeconomic status is different than the microbiota of people who live in high-income countries.

The human microbiota exhibits high interindividual variability [14,15]. However, some populations share a similar microbiota that distinguishes them from others. For instance, the intestinal microbiota of adults and children from the Amazonas State of Venezuela and rural Malawi have marked differences regarding phylogenetic composition and functional gene groups than that of adults and children from the United States (US), the former being more diverse [16]. There are also marked differences between individuals from native societies in Papua New Guinea and US residents, the microbiota of the former being more diverse, with lower inter-individual variation and heterogeneous abundance profiles, composed by different taxa [17]. Consistent with this, the gut microbiota of Malaysian individuals is more diverse than that of New York City inhabitants, exhibiting a particular bacterial community structure [18]. Interestingly, the gut microbiota genetic potential is more similar between two LMICs than when comparing any of them with USA samples [19]. Thus, the gut microbiota of people from LMICs differs from that of individuals living in industrialized, high-income countries.

Another aspect of LMICs is that the gut microbiota composition of native populations from different continents (e.g., the Hadza hunter-gatherers of western Tanzania and the Yanomami and Guahibo of Venezuela) is characterized by taxa that are absent or less frequent in industrialized populations (i.e., Prevotellaceae, Spirochaetaceae, Succinivibrionaceae), whereas Bacteroidaceae and Verrucomicrobia are more abundant in individuals from industrialized countries [20]. When comparing the intestinal microbial composition of children from Italy with that of children from the Mossi ethnic group living in a rural village in Burkina Faso, the latter has higher richness and biodiversity, is enriched in Bacteroidetes (e.g., *Prevotella* and *Xylanibacter*), and has a lower proportion of Enterobacteriaceae. Moreover, stool samples taken from Mossi children had higher levels of short-chain fatty acids (SCFA) [21]. Available evidence points towards diet as a major determinant of these differences, but it is not the only contributing factor. A comparison among the gut microbiota of four Himalayan populations (three of which transitioned from hunting-gathering to farming, whereas the other one is still a foraging population) showed a spectrum of microbial composition with foragers at one end and the farmers who transitioned earlier at the other. In addition, the intestinal microbiota of the rural Himalayan populations is distinguishable from that of American individuals, with farming communities showing most similarities [22]. The gut microbiota of rural villagers from El Salvador and Peru slum inhabitants, when compared to that of people from the US, Malawi, Venezuela, and other populations of Peru, exhibits clustering by lifestyle (i.e., farming/hunting-gathering, peri-urban, or industrialized urban) [23].

Interestingly, migration from South Asia to North America modifies the intestinal microbiota, which may increase the risk for metabolic diseases such as obesity [24,25]. Gut microbiotas from Thai individuals who immigrated to the US and their US-born offspring showed a loss of microbial diversity and native bacterial taxa compared with their Thai-based counterparts. This effect was more pronounced with longer duration of US residence and in second-generation immigrants. Migration was also associated with long-term changes in the relative abundances of dominant taxa in the gut microbiota, particularly a shift from *Prevotella* to *Bacteroides*, and with functional alterations involving loss of plant fiber-degrading glycoside hydrolases (which were mainly produced by *Prevotella copri*). These changes began as soon as 6–9 months post-immigration and were partly explained by dietary acculturation [24]. Similar findings were reported in Canadian immigrants born in South Asian countries and their children, showing that the time spent in Canada was associated with changes in the gut microbiota characterized by a decrease in *P. copri* and *Dialister succinatiphilus* abundance and an increase in *Bacteroides* and *Dialister invisus*. These compositional alterations coincided with differences in gene families related to carbohydrate degradation and SCFA production [25].

It has also been observed that, within a given country, people with lower socioeconomic status have different microbiota. In a tropical country like Indonesia, schoolchildren living in an urban area have different gut microbiota depending on their socioeconomic status. Although some taxa are present in all children and bacterial richness is similar, some genera (e.g., *Bifidobacterium*, *Lactobacillus*, *Prevotella*, and *Escherichia-Shigella*) are differentially represented in children with high vs. low socioeconomic status. The gut microbiota of children with low socioeconomic status is significantly more diverse; in addition, the overall intestinal microbial composition of children with high socioeconomic status is associated with nutritional status, as measured by body mass index-for-age [26]. Furthermore, some studies including children from low-income countries have shown an association between intestinal microbiota composition and diarrhea, raising the possibility that some of the bacterial taxa identified in these studies might modify the risk for this condition [27,28].

Besides bacteria, the characteristics of other microbial communities such as intestinal protozoa in people from LMICs are expected to be different. However, research in this matter goes beyond the scope of this review.

**Figure 1:**
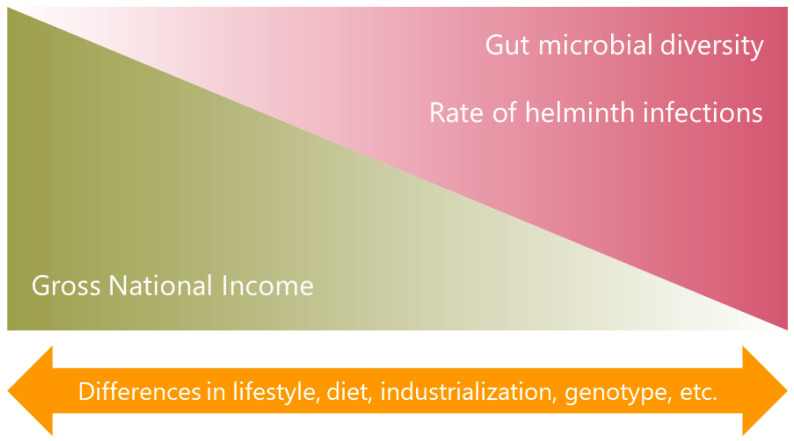
National income and diversity of the gut microbiota. The higher diversity of the intestinal bacterial communities among populations from LMICs coincides with a high rate of soil-transmitted helminth infections in those countries, suggesting that parasites could be involved in these findings together with other factors.

In summary, a common finding is that people from LMICs or native populations, have more microbiota diversity (Figure 1). However, more studies are needed comparing the microbial profile between individuals from LMICs and those from high-income countries or between people of high and low socioeconomic status within the same country or region, focusing on unraveling the factors that underlie the observed differences. These data suggest that lifestyle and industrialization with its multiple consequences, including its potential effects on helminth infections, play a significant role in shaping the microbiota.

## 3. Effects of the Microbiota on Helminth Infections

### 3.1. Animal Models

This section analyzes the influence of the microbiota on the risk and pathogenesis of intestinal parasitic infections in animals. One aspect is the need for microbiota at the beginning of an infection. For example, in a murine model of *Heligmosomoides polygyrus* infection, parasites infecting germ-free mice had reduced fecundity and a distinct gene expression profile, including differential expression of genes with putative antimicrobial properties. These mice exhibited higher T helper type (Th2) responses in the small intestine and reduced forkhead box P3 (Foxp3)^+^ RAR-related orphan nuclear receptor (ROR)γt^+^ regulatory T cells than mice with commensal microbiota, suggesting that the microbiota drives immunomodulatory pathways that benefit the parasites [29].

Also, BALB/c mice are resistant to *H. polygyrus* infection, but there is interindividual variation in their ability to clear these parasites [30]. The burden of *H. polygyrus* infection correlates positively with the abundance of Lactobacillus/*Lactococcus* species in the duodenum of these mice, but not with other bacteria. Both the parasite burden and the abundance of Lactobacillus/*Lactococcus* correlate positively with parasite specific Th17 and regulatory T cell (Treg) responses as well. *Lactobacillus taiwanensis* seems to be the main driver of this association because administration of this microbe to BALB/c mice enhances the helminth establishment and increases Treg frequencies [30].

Another interesting example is the infection of mice with *Trichuris muris*. The eggs of this parasite need direct contact with commensal bacteria (e.g., *Escherichia coli*) to hatch, probably through bacterial surface adherence molecules such as type 1 fimbriae [31]. In addition, this helminth requires the presence of the host microbiota (including *Bacteroides thetaiotaomicron*) to successfully infect, and through the changes it exerts on the host gut microbiota, limits ensuing infections by other individuals of the same species [32]. Similar to *H. polygyrus*, a species of lactobacilli (*Lactobacillus casei*) enhances susceptibility to *T. muris* infection [33].

There is also evidence of the opposite effect, where the microbiota is a resistance factor for the infection. For instance, antibiotic treatment impaired the expulsion of *Trichinella spiralis* triggered by beta-glucans, indicating that such an effect is dependent on the microbiota, and this seems to be mediated by increased mucus production and secretion, rather than by type 2 immunity. Beta-glucans induced an expansion of Verrucomicrobia, including the genus *Akkermansia*. More specifically, *Akkermansia muciniphila* enhances the production of mucus in the gut and reduces the *T. spiralis* worm burden via toll-like receptor 2 (TLR2) [34].

**Figure 2:**
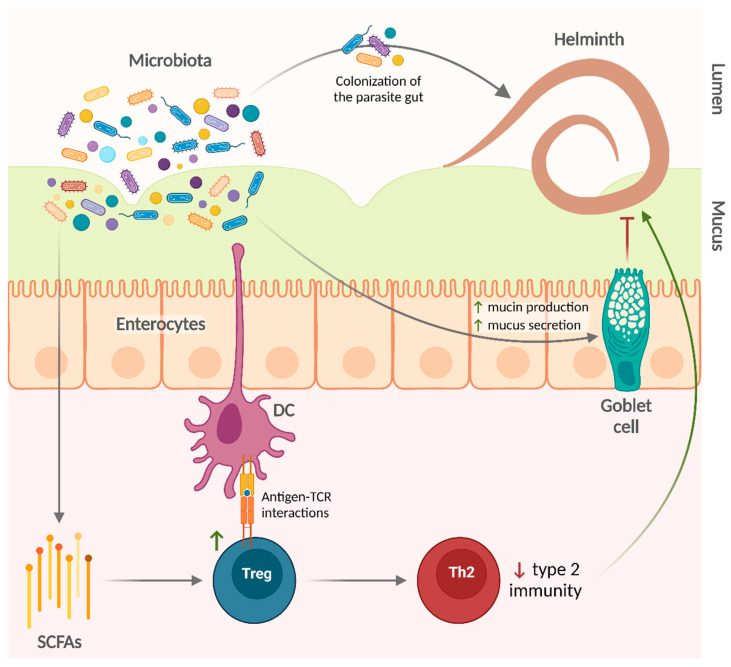
Mechanisms underlying the effects of the microbiota on helminth infections. Commensal bacteria can favor the establishment of helminth infections or enhance worm expulsion through different mechanisms. In addition, the helminth gut is colonized by bacteria from the host microbiota, from which it forms its own. DC, dendritic cell; SCFAs, short-chain fatty acids; TCR, T cell receptor; Th2, type 2 helper T cell; Treg, T regulatory cell.

Taken together, these studies indicate that the microbiota is involved in the regulation of gut colonization by helminth parasites (Figure 2). Some helminths require the host microbiota to establish an infection and to acquire their own microbiota. Specific bacterial taxa underlie the intrinsic susceptibility of the host to certain parasitic infections, at least partially by an expansion in the Treg population. However, commensal bacteria can also help in the control of helminth infections.

### 3.2. Humans

Some studies in humans provide information about the impact of the host microbiota on helminth infection, both limiting or enhancing it. Individuals that are able to self-clear helminth infections have more similar microbiota assemblages compared to those who remain infected [19]. Specific bacterial taxa (e.g., *Subdoligranulum*) might contribute to resistance to the infection in the self-clearing individuals, while the genus *Ruminococcus* is associated with infection at baseline and prevention of parasite clearance [19]. In agreement with this, it has been reported that *Ruminococcus*, *Dorea*, and two other microbial operational taxonomic units (OTUs) belonging to the Clostridiales order were positively associated with *T. trichiura* egg burden, whereas *Prevotella* and Lachnospiraceae showed an inverse association [35].

Thus, the microbiota might influence the susceptibility to helminth infection in humans, as well as its course and outcomes. Given the limited number of studies, validation across human populations is needed. As described above, microbial gut communities are needed to stablish successful helminth infections in animals; however, their importance in humans has not been yet fully demonstrated. Further baseline and prospective microbiota analysis would help to expand our knowledge on the microbiota-to-helminth effects in humans.

## 4. Influence of Helminth Infections on the Microbiota

There are several lines of evidence suggesting that helminth infections modify the intestinal microbiota. Helminth excretory/secretory products (ESPs) contain several antimicrobial peptides (AMPs) with both lethal and non-lethal effects that can directly modify gut bacterial communities [29,36,37]. Although there is no experimental evidence of this direct effect on the whole microbiota composition, it is likely that this mechanism acts together with other pathways to convey the helminth-induced changes in the microbiota that are observed in both humans and animal models.

### 4.1. Animal Models

Animal models—including rodents, pigs, fish, and others—have been very useful for studying the alterations of the microbiota associated with parasite colonization and their underlying mechanisms. One important aspect of the helminth–microbiota relationship is the role of the host genetic background. Using a fish model (*Gasterosteus aculeatus*) it was observed that the influence of a helminth (*Schistocephalus solidus*) on the gut microbiota was dependent on the sex and autosomal genotype of the host. Clostridiales were less abundant in infected than uninfected males, whereas no differences were observed between infected and uninfected females, showing a statistically significant sex-infection relationship. As for the genetic factors implicated, it was found that some quantitative trait loci for the abundance of different bacterial orders were dependent on infection status [38]. *S. solidus* is not an intestinal parasite but transits to the peritoneal cavity within hours after ingestion to establish its infection without directly contacting the gut microbiota. Therefore, the mechanisms underlying these findings remain to be studied.

It has been suggested that helminths alter the gut microbiota composition in a species-specific way [39]. Thus, the effects of different helminth species on the microbiota of some animal models will be analyzed. Infection with *H. polygyrus* increases Lactobacillaceae/*Lactococcus* species abundance in the duodenum and ileum of a susceptible mice strain (i.e., C57BL/6), but not in a resistant strain (i.e., BALB/c) [30,40], suggesting that these effects are dependent on the host’s intrinsic susceptibility to the parasite. Similarly, a higher abundance of Lactobacillaceae has been reported in *Nippostrongylus brasiliensis* infection [41], but *H. polygyrus* infection also increases the abundance of other taxa, as was reported for an unidentified species belonging to the Peptostreptococcaceae family [42], and the gram-negative Enterobacteriaceae and Bacteroides in multiple intestinal sites [43].

In another setting, a worm burden-independent reduction in gut microbial diversity has been observed in pigs infected by *A. suum* [44]. This helminth also alters the composition and metabolic potential of the porcine gut microbiota [44,45] and, by itself or in combination with dietary factors such as polyphenols, affects the levels of short chain fatty acid (SCFA) in the intestine, most likely through changes in the microbiota [44,45].

*T. muris* and *Trichuris suis* are also known as the mouse whipworm and porcine whipworm, respectively. They are very similar to the human whipworm, *T. trichiura*. Some whipworm-associated gut microbial changes have been consistently identified in mice and humans [46]. In mice, chronic infection with *T. muris* reduces the alpha diversity of the gut microbiota and changes its composition, with a reduction in the diversity and abundance of Bacteroidetes, such as *Prevotella* and *Parabacteroides*, and an increased abundance of *Lactobacillus*. These changes were not observed at the beginning of the infection but established over time and some of them were partially reversed by anthelmintic treatment, suggesting that the presence of the parasite is needed to maintain these modifications [47,48]. The association of some microbial taxa (e.g., *Prevotella*) with whipworm infection does not have a consistent direction of effect; for example, some operational taxonomic units (OTUs) belonging to this genus exhibit positive associations whereas others show an opposite relationship in the same experimental model [46].

*T. suis* has also been associated with changes in the porcine colonic microbiota, altering the abundance of some taxa, such as Proteobacteria and Deferribacteres, and significantly affecting 13% of genera, including *Succinivibrio*, *Mucispirillum*, *Fibrobacter*, and *Ruminococcus* [49]. In another study, some of the compositional changes were dependent on the worm burden: *Campylobacter* abundance was increased three-fold in infected pigs with worms, with considerable difference compared to ‘worm-free’ infected pigs [50]. This helminth also induces functional alterations in the gut microbiota, comprising various metabolic pathways like carbohydrate and lysine metabolism [49,50]. Moreover, dietary inulin enhances the modifications induced by *T. suis* in some putatively beneficial taxa such as *Prevotella* [51].

It is worth mentioning other aspects of the influence of helminth on the microbiota. For example, in a murine model of *H. polygyrus* infection, it was observed that the changes in the bacterial communities are influenced by the “starting point” of the host gut microbiota [42]. Besides, *T. muris* can promote bacterial invasion into inner layers of the intestinal epithelium [52], with the potential modification of the microbiota composition, an aspect that deserves further investigation.

Regarding the role of the immune responses on the helminth–microbiota relationships, it was found that pre-conceptional maternal helminth infection with *N. brasiliensis* modifies breastmilk and offspring fecal microbiota, as well as the offspring’s inherent immunity. Interestingly, microbiota programming in pups begins in utero and continues during breastfeeding; however, there was no evidence of direct transfer of microbes from breastmilk to infant gut [53]. Since the infection was cleared before mating by ivermectin treatment, vertical transmission of parasites was highly unlikely. This study suggests a role for the immune system in the helminth-induced changes in the microbiota.

**Figure 3:**
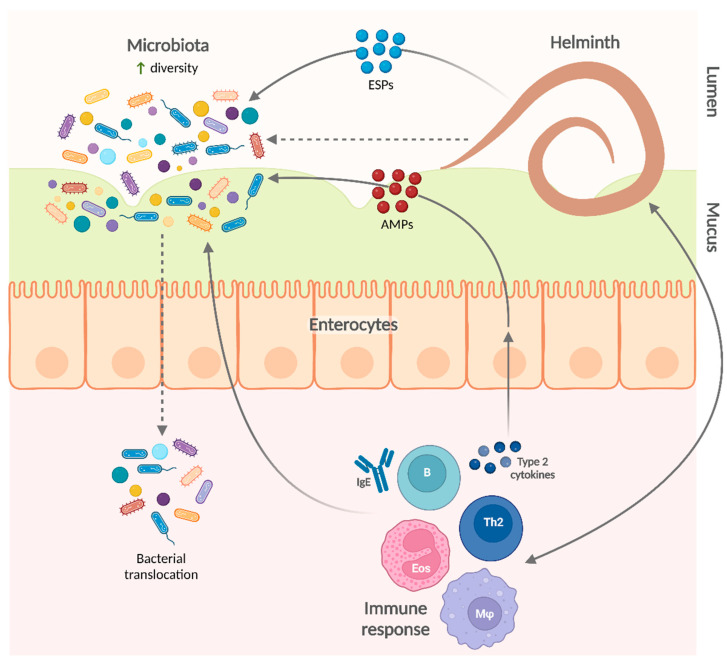
Mechanisms involved in the influence of helminth infections on the microbiota. Helminths can modify the gut microbiota composition by several mechanisms and are generally associated with a more diverse gut microbiota. AMPs, antimicrobial products; B, B cell; Eos, eosinophil; ESPs, excretory-secretory products; IgE, immunoglobulin E; Mφ, macrophage; Th2, type 2 helper T cell.

In summary, animal models have provided insights into the impact of helminth parasites on the microbiota (Figure 3). Additional experimental work will further elucidate the mechanisms underlying these effects.

### 4.2. Humans

Helminths have been regarded as important contributors to gut microbiota variation in humans [35]. There are several epidemiological studies showing these effects in various populations and some of them will be reviewed here.

The microbiota of helminth-infected individuals exhibits high diversity, especially in polyparasited individuals [54,55]; which supports a role of helminth infections in the particularities of the microbiota found in people living in LMICs. Apart from an increased richness and diversity, the gut microbiota of helminth-infected individuals in Philippines exhibits a higher abundance of *Faecalibacterium* that increases significantly with polyparasitism [55]. In addition, compared to uninfected controls or those infected only with protozoans, the fecal microbiota of young children from Medellín (Colombia) infected with *A. lumbricoides* (together with other parasites) show enrichment of Prevotellaceae and *Prevotella* at the expense of Bacteroidaceae and *Bacteroides*, at the family and genera level, respectively. This was also observed among children infected with *Giardia intestinalis* [56]. However, since children with ascariasis were also infected by *Giardia intestinalis*, it is not clear whether these changes are shared between these parasites or driven by giardiasis.

Other studies have also shown helminth-associated alterations in the microbiota composition, although with different outcomes.

Gut microbiota of people living in an area with a low rate of intestinal helminth colonization have a higher abundance of *Bacteroides*, whereas *Faecalibacterium* and *Prevotella* are characteristic of people from a helminth-endemic area [57].

Conversely, among Indonesian schoolchildren of low socioeconomic status, *Prevotella* and unclassified Lachnospiraceae are more abundant in those without helminth infection; whereas children harboring helminths have a higher relative abundance of *Lactobacillus*, *Olsenella*, *Enterorhabdus*, and *Morgibacterium* [26]. Supporting this work, taxa belonging to the Lachnospiraceae family were less abundant among individuals infected with soil-transmitted helminths in India [58]. In addition, a survey from Indonesia and Liberia found that *Olsenella* and Lachnospiraceae were, respectively, positively and negatively associated with infections by helminths such as *A. lumbricoides*, *N. americanus*, and *T. trichiura* [19]. These contrasting findings regarding some taxa could be related to the studied population, although other factors may be involved.

An interesting study that includes several aspects of helminth infections was done by Jenkins et al. [59], who found that there are differences in microbial diversity and bacterial family representation between individuals infected with helminths (including *Ascaris*, *Trichuris*, and hookworms), uninfected subjects, and people who had received regular prophylactic anthelmintic treatment. The intestinal microbiota of people without helminth infection was characterized by a lower beta diversity and more abundance of bacteria belonging to the Leuconostocaceae family. Verrucomicrobiaceae and Enterobacteriaceae were increased among subjects with helminthiasis, whereas Bacteroidaceae was more represented in the anthelmintic treatment group [59]. Although no significant differences in alpha diversity or species richness were found in this study, another survey showed that helminth infection is associated with greater alpha diversity and, particularly, greater species richness. Paraprevotellaceae, Mollicutes, Bacteroidales, and Alphaproteobacteria were increased among individuals with helminth infections, whereas *Bifidobacterium* was more abundant in the helminth negative group [18].

It is very difficult to detect the effects of individual helminths on human microbiota; however, some authors have explored this important aspect. *T. trichiura* has been considered one of the main parasites influencing the gut microbial composition in humans [54]. One study analyzed the effect of whipworm infection in both humans and mice, with some genera showing positive associations with infection, including *Escherichia*/*Shigella*, *Prevotella* 2 and 9, *Streptococcus* and *Bacteroides*, and other negatively associated, among them *Blautia* and other genera from the family Lachnospiraceae [46]. In contrast, infection by *T. trichiura* alone did not significantly affect the gut microbiota of Ecuadorian schoolchildren, but changes in the bacterial community profiles were observed in mixed infections with *T. trichiura* and *A. lumbricoides*—including a negative association with *Clostridium sensu stricto* and an unusually high proportion of *Streptococcus* in the guts of infected individuals [60]. This suggests that the type of helminth, as well as the combination of helminths, have different effects on the microbiota.

Other helminths have also been individually investigated. In a case report of pediatric hyper-infection with the nematode *Strongyloides stercoralis,* it was shown that fecal samples taken during or one month after infection have higher alpha diversity and are more similar to each other in terms of beta diversity and composition, compared with those taken two months after infection or from healthy controls [61]. Besides, some studies have shown that infections by trematodes—such as schistosomes, *Clonorchis sinensis*, and *Haplorchis taichui*—are associated with changes in the gut microbiota (e.g., higher abundance of *Bacteroides*) [62,63,64,65].

Comparisons of the microbial communities before and after deworming provide valuable information about the potential effects of helminths on the microbiota, although there are contrasting findings. Among parasite-infected individuals from an *A. lumbricoides*- and *N. americanus*-endemic area in Kenya, Enterobacteriales were more common at baseline, whereas Clostridiales were more common 3 months following albendazole treatment [66]. However, in Malaysia, Ramanan et al. observed that anthelmintic treatment reduced alpha diversity and the relative abundance of bacteria of the order Clostridiales, while increasing those belonging to Bacteroidales [57]. Other studies shed light on the effects of helminth infection on Clostridiales. In individuals from Indonesia and Liberia, seven out of the 12 taxa increased with helminth infection belonged to the Firmicutes phylum, including four genera from the Clostridiales order [19]. Also, as described before, *T. trichiura* egg burden was positively associated with four OTUs belonging to the Clostridiales order [35]. These results suggest that helminths favor the expansion of Clostridiales in the gut; however, this association deserves further exploration, especially considering the potential homeostatic and health-promoting effects of some members of this taxon [67,68,69,70].

In conclusion, helminth parasites change the human microbiota upon infection in terms of diversity and composition. Consistent with data from LMICs, helminth infection is generally associated with an increase in microbial diversity, although these findings are not always replicated. Besides, the specific effects of helminthiasis on microbial composition differ among the studies, which could be attributed to dissimilarities in population, age, geographic location, lifestyle, or technical factors. However, it is also likely that individual parasites affect the microbiota in distinct ways with species-specific mechanisms.

## 5. Biological and Clinical Consequences of the Modifications Exerted by Helminth Infections on the Microbiota

### 5.1. Animal Models

**Figure 4:**
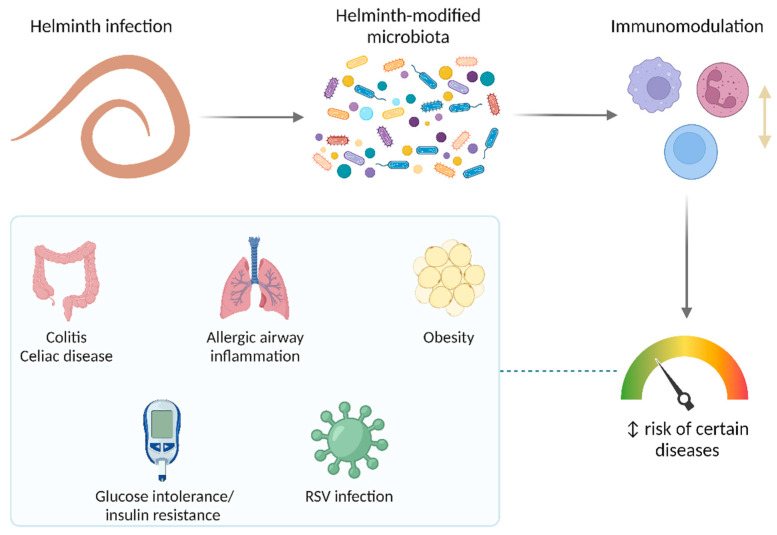
Impact of helminth-induced microbiota modifications on the risk of certain diseases. Helminth infections can modify the risk of some diseases; in most cases, reducing it. A microbiota-mediated regulation of the immune response is thought to be a key player. RSV, respiratory syncytial virus.

It is well known that several parasites modulate the host immune response through various mechanisms. Since helminths also modify the microbiota of their hosts, it is expected that, by these mechanisms, they can also impact health and disease. In recent years, the microbiota-mediated effect of helminthiasis on diseases has emerged as an interesting and promising area of research (Figure 4).

*Nod2* deficiency in mice induces inflammation in the small intestine in a microbiota-dependent way, mainly mediated by *Bacteroides vulgatus* [71]. Two helminths (*T. muris* and *H. polygyrus*) prevented the intestinal abnormalities seen in *Nod2*^-/-^ mice by limiting colonization by *B. vulgatus* and increasing Clostridiales, effects that seem to be mediated by a type 2 immune response [57]. Similarly, changes in the microbiota associated with *Hymenolepis diminuta* colonization, like an increase in the abundance of Clostridiales (including Lachnospiraceae), are critical for the protection from dinitrobenzene sulfonic acid-induced colitis in mice [72]. This effect is mediated, at least in part, by SCFA because feces from mice colonized by this tapeworm have increased levels of these compounds, which is prevented by antibiotic treatment. In addition, a bacteria-free filtrate of feces from *H. diminuta*-colonized mice protects against colonic inflammation, whereas this is not observed in mice lacking a receptor for SCFA called free fatty acid receptor (FFAR)2 [72]. However, there are experiments with opposite results; for example, it has been found that *H. polygyrus* exerts Th2-mediated changes in the gut microbiota (i.e., expansion of Bacteroidetes and reduction of Firmicutes) that exacerbate the bacterial colitis produced by *Citrobacter rodentium* [73].

Regarding other diseases, Zaiss et al. showed that chronic infection with *H. polygyrus* attenuates allergic airway inflammation in mice, which requires parasite-induced changes in the microbiota towards a more SCFA-producing profile characterized by an increase in Clostridiales (particularly, Lachnospiraceae). The SCFA receptor FFAR3 in the host was required for the induction of anti-inflammatory pathways and the observed protective effect [74]. Also, the enhanced ability of *H. polygyrus*-modified microbiota to produce SCFA underlies its ability to induce resistance to obesity and glucose intolerance [75], which seems to be the same for *T. spiralis* [76]. Other studies suggest that *N. brasiliensis* and *Strongyloides venezuelensis* improve insulin sensitivity in mice, likely through their impact on the microbiota [77,78]. The alteration of the gut microbiota exerted by *H. polygyrus*—and probably other helminths as well—is determined by a type 2 immune response involving M2 macrophages [75,76,77].

In addition to non-communicable diseases, the helminth-induced changes in the microbiota could also protect against infections. For instance, *H. polygyrus* reduced viral load, inflammation, and disease manifestations in a murine model of respiratory syncytial virus infection. A microbiota-dependent innate immune response involving type I interferons was required for this protective effect [79].

### 5.2. Humans

Studies looking for microbiota-mediated effects of helminth infections on diseases in humans are even less conclusive. However, important advances have been obtained. For example, experimental infection with the hookworm *N. americanus* improves gluten tolerance in celiac disease patients subjected to escalating dietary challenges [80]. Changes in the microbiota are a potential mediator of this beneficial effect of hookworm infection. Even though the experimental infection with *N. americanus* seems to have little impact on the human fecal microbiota within a short window of time, it is associated with a significant increase in microbial richness several months after the infection [81,82]. But these studies have limitations; they were conducted in volunteers with controlled celiac disease who have been on a gluten-free diet for at least five years and the preexistence of this inflammatory condition might have imprinted the gut microbiota towards a relative insensitivity to helminth modifications. The results of these hookworm infection trials could also be partially explained by the use of fecal samples for microbiota analyses, since this may not reflect the actual changes that are observed in different regions of the intestinal tract. Actually, when analyzing duodenal mucosally-associated microbiota, increased alpha diversity and abundance of Bacteroidales were observed at 24 weeks post-infection with *N. americanus* (in addition to gluten challenges) [83]. These findings are in line with a role of the microbiota in the improvement of gluten tolerance; but more conclusive evidence is needed.

## 6. Discussion

Helminth parasites and the microbiota have coexisted within their hosts for millions of years [6], establishing a trilateral interplay. Their longstanding interaction with humans and our ancestors throughout evolution has earned them the title of “old friends”, since they have played a role in shaping our immune system physiology. Nonetheless, the demonstrable negative impact helminths have on human health outweighs any potential benefits of these infections. In fact, controlling helminthiases must be a public health priority. We can keep their important evolutionary legacy by using their immunomodulatory molecules. Since some of the benefits of helminths might be mediated by the microbiota, this review contributes to consolidate our understanding on this matter.

Humans and other animals are colonized since birth with commensal bacteria and can encounter helminths throughout their life, providing a niche for interdomain exchanges. In this review, the evidence regarding these relationships was analyzed. In brief, it can be concluded that: first, the microbiota of individuals from developing countries is more diverse than that found in industrialized countries; second, the microbiota influences the presentation and evolution of some helminth infections and, vice versa, helminth infections alter the microbiota diversity and composition; and third, the interactions among microbiota, helminth parasites, and the host seem to be important for the evolution of some diseases.

People living in LMICs have a high chance of being infected by parasites. As expected, the microbiota of LMICs populations is different from that of high-income countries, likely reflecting an effect of parasites, although several other factors play a role in shaping the microbiota. Hence, the evidence from the studies conducted in subjects from LMICs, albeit revealing, is only suggestive of the great magnitude of parasite–microbiota crosstalk, although research in helminth-infected humans and animal models has allowed us to improve our understanding of this matter.

Observations in LMICs-based populations and infected individuals indicate that helminths promote a more diverse intestinal microbiota. This is especially interesting given that microbial diversity has been linked to a reduced risk of chronic inflammatory diseases such as allergic conditions, obesity, and inflammatory bowel disease [84,85,86,87,88,89]. In fact, some epidemiological studies have shown a lower rate of these diseases in developing countries [90,91,92,93]. A helminth-induced increase in the abundance of health-promoting microbes is a plausible mechanism underlying these protective effects. Several basic studies have shown that, in some cases, helminths promote the expansion of SCFA-producing bacterial taxa, modifying the metabolic potential of the microbiota. The helminth-induced increase in levels of SCFA is observed across diverse helminth and host species, including pigs infected with *A. suum* and humans infected with *N. americanus* [74]. In addition, some of the helminth-induced effects might be associated with an increased abundance of Lactobacillaceae. Bacteria belonging to this family have been shown to attenuate inflammation and regulate immune response through different mechanisms—including Tregs, inhibition of proinflammatory cytokines, and blockage of inflammatory signaling pathways [94,95,96,97,98].

Regarding the effects of microbiota on helminth infections, there are also additional basic experimental findings supporting the studies that were analyzed in this review. Gut bacterial communities can promote the development of Tregs—via the production of SCFA and antigen-T cell receptor interactions [99,100,101,102]—that regulate type 2 immune responses [103] in favor of helminths. On the other hand, bacteria such as *A. muciniphila* degrade mucin and produce SCFA that in turn could provide energy to mucin-producing goblet cells [104,105,106], thus enhancing mucus production and facilitating worm expulsion. Gut bacterial communities are a complex mixture of several microbial species. Nonetheless, specific commensal species are sufficient to promote (e.g., *L. taiwanensis* and *B. thetaiotaomicron*) or limit (e.g., *A. muciniphila*) infection by certain helminths.

SCFA (e.g., acetate, butyrate, and propionate) are lipid compounds produced mainly by the gut microbiota from the metabolism of dietary fiber. Bacteria belonging to the phylum Bacteroidetes mostly produce acetate and propionate, whereas Firmicutes mainly produce butyrate [104]. Particularly, species within the Ruminococcaceae and Lachnospiraceae families (order Clostridiales) are among the most important producers of butyrate [104]. Interestingly, these two families were enriched among asthmatic subjects with low specific IgE levels against *A. lumbricoides* [11]. Helminths also produce SCFA themselves [107]; these fatty acids can act through their receptors (FFARs) and are also known histone deacetylase inhibitors, allowing them to exert many immunomodulatory properties [108]. As mentioned before, these metabolites stimulate the development of Tregs, probably by an enhanced histone H3 acetylation in the regulatory regions of *Foxp3* locus [100,109]. Since there are reports suggesting that *A. lumbricoides* infection induces epigenetic changes on the host’s immune response genes [10,110], it would be interesting to define if these epigenetic modifications are direct effects from the parasite or mediated by their interactions with the microbiota.

The impact of helminths on the microbiota could be explained by multiple mechanisms. Helminth products (including AMPs) could directly affect the diversity and composition of the microbiota. For example, *A. suum* ESPs contain several AMPs—such as lectins, cecropins, and members of the *A. suum* antibacterial factor family—which inhibit bacterial growth, impair biofilm formation, or neutralize bacteria by other mechanisms (e.g., agglutination) [111,112]. Helminth ESPs also include extracellular vesicles, which contain putative AMPs and are thought to be important mediators of the effects of helminths on the microbiota [113,114]. In addition, type 2 cytokines produced during helminth infection also induce host cells to produce AMPs like small proline-rich protein 2A, which shapes intestinal microbiota composition and protects against helminth-induced bacterial invasion of intestinal tissue [115].

Even though the available evidence has helped to understand some aspects of the helminth–microbiota interactions, the heterogeneity among studies prevents from drawing more general conclusions. Several factors could explain discrepancies between studies, among them, the species of the host, interindividual variation, interpopulation variation, pre-existing microbiota, type of helminth, duration of the infection (i.e., acute vs. chronic), parasite burden, and the type of sample (e.g., fecal) used for analysis. In addition, different OTUs within a given bacterial taxon can exhibit opposite directions of association with helminth infection, which can confound the associations results when analyzing higher taxa.

## 7. Perspectives

Helminth–microbiota interactions have gained increasing interest among the scientific community, bringing forward many questions that have been the subject of considerable research, especially in the last decade. Nevertheless, a great number of questions remain unanswered and await further investigation.

Several studies report helminth–microbiota associations and, in the future, research in this field is expected to grow significantly in LMICs. In many cases it is not possible to elucidate whether these associations are due to helminth-to-microbiota effects, microbiota-to-helminth effects, or both. Hence, more findings clarifying the directionality of these associations is needed. In addition, recent experimental data suggest that, in the future, analysis of the microbiota could predict the outcomes of some helminth infections such as schistosomiasis [116]. Moreover, oncoming data from experimental research would help increase our understanding of the mechanisms of the helminth–microbiota interactions, including studies that elucidate the involved host’s genetic factors.

## Data Availability

Not applicable.
